# Household Dengue Prevention Interventions, Expenditures, and Barriers to *Aedes aegypti* Control in Machala, Ecuador

**DOI:** 10.3390/ijerph14020196

**Published:** 2017-02-16

**Authors:** Naveed Heydari, David A. Larsen, Marco Neira, Efraín Beltrán Ayala, Prissila Fernandez, Jefferson Adrian, Rosemary Rochford, Anna M. Stewart-Ibarra

**Affiliations:** 1 Colorado School of Public Health, University of Colorado Denver, Aurora, CO 80045, USA; rosemary.rochford@ucdenver.edu; 2 Center for Global Health and Translational Science, State University of New York Upstate Medical University, Syracuse, NY 13210, USA; pipita_fernandez@hotmail.com (P.F.); jeff.adn@gmail.com (J.A.); amstew01@gmail.com (A.M.S.-I.); 3 Department of Public Health, Food Studies and Nutrition, Syracuse University, Syracuse, NY 13244, USA; dalarsen@syr.edu; 4 Center for Research on Health in Latin America (CISeAL), Pontificia Universidad Catolica del Ecuador, Quito 170170, Ecuador; MVNEIRA@puce.edu.ec; 5 Universidad Técnica de Machala, Machala 070102, Ecuador; felixbeltran57@hotmail.com

**Keywords:** *Aedes aegypti*, mosquito control, economic cost, dengue fever, KAP, Ecuador

## Abstract

The *Aedes aegypti* mosquito is an efficient vector for the transmission of Zika, chikungunya, and dengue viruses, causing major epidemics and a significant social and economic burden throughout the tropics and subtropics. The primary means of preventing these diseases is household-level mosquito control. However, relatively little is known about the economic burden of *Ae. aegypti* control in resource-limited communities. We surveyed residents from 40 households in a high-risk community at the urban periphery in the city of Machala, Ecuador, on dengue perceptions, vector control interventions, household expenditures, and factors influencing purchasing decisions. The results of this study show that households spend a monthly median of US$2.00, or 1.90% (range: 0.00%, 9.21%) of their family income on *Ae. aegypti* control interventions. Households reported employing, on average, five different mosquito control and dengue prevention interventions, including aerosols, liquid sprays, repellents, mosquito coils, and unimpregnated bed nets. We found that effectiveness and cost were the most important factors that influence people’s decisions to purchase a mosquito control product. Our findings will inform the development and deployment of new *Ae. aegypti* control interventions by the public health and private sectors, and add to prior studies that have focused on the economic burden of dengue-like illness.

## 1. Introduction

Febrile illnesses transmitted by the *Aedes aegypti* mosquito—such as Zika, dengue, and chikungunya—present a rapidly increasing public health problem in tropical and subtropical regions, where a large proportion of the world’s population are at risk of disease [1]. Low-income countries are particularly vulnerable to the social and economic impacts of these emerging epidemics due to limited resources in the public health sector and at the household-level to prevent and manage the disease [2]. Prior studies estimate that the economic burden of dengue infections in the Americas costs US$2.1 billion per year on average (in 2010 US dollars), with a range of US$1–4 billion [3]. Initial estimates of the short-term economic impact of the Zika virus epidemic for 2016 in the Latin American and the Caribbean region are US$3.5 billion [4]. Control of *Ae. aegypti* mosquitoes through chemical and biological targeting and management of larval habitat (e.g., containers with water) remains the principal means of preventing and controlling dengue, chikungunya, and Zika outbreaks. However, relatively little is known about the economic burden of and barriers to household-level mosquito control in resource-limited communities. 

In Ecuador, febrile illnesses transmitted by *Ae. aegypti* have replaced malaria as the most prevalent-mosquito borne diseases. Over a five year period (2010 to 2014), 72,060 cases of dengue were reported in the country, as compared to 1138 cases of malaria [5]. Dengue is hyper-endemic in Ecuador’s lowland coastal region, where new cases present every year, with peak transmission during the rainy season (February to May), and sporadic transmission the rest of the year. The first cases of locally acquired chikungunya were reported in Ecuador at the end of 2014, resulting in over 33,000 cases in 2015. The first cases of Zika were reported in the country on 7 January 2016, and to date (6 October 2016) 2695 suspected cases of Zika have been reported [6]. The primary vectors for these diseases are *Ae. aegypti* and *Ae. albopictus*; however, *Ae. albopictus* has not been reported in Ecuador. Ongoing surveillance studies in Machala indicate that the true burden of these vector-borne diseases is much higher than reported due to a high proportion of asymptomatic or mild cases and limited access to laboratory diagnostics (Stewart-Ibarra et al. in prep).

Previous studies in Machala indicate that local social-ecological conditions (e.g., poor housing conditions, piped water infrastructure, demographics of the heads of households) influenced *Ae. aegypti* abundance and the presence of dengue [7,8,9]. Community members in two proximate urban areas specified economic factors as a key barrier to dengue prevention, including low household income, employment, type of housing, the cost of water storage (e.g., cisterns), and the cost of vector control [9]. However, no prior studies have estimated the economic burden of *Ae. aegypti* control in these communities.

In 2015, the Ministry of Health (MoH) in Ecuador began a transition from a vertical vector control program to a horizontal decentralized program with integrated management strategies in *Ae. aegypti* endemic regions. The MoH typically conducts focal fumigation in and around the households of individuals with dengue, chikungunya, or Zika. In these areas, environmental fogging is conducted from trucks, and household visits are performed by MoH field workers to eliminate larval habitats by overturning containers with water and treating stored water with larvicides (*Bti* and temefos). The government bears the burden for costs of dengue prevention and vector control, thus the interventions are free from the perspective of the household. However, in the peripheral areas of Machala, public health personnel are not able to effectively implement these vector control strategies due to limited resources. Therefore, in the absence of effective interventions by government institutions, it has become imperative that families invest household resources to protect themselves against dengue fever and other mosquito-borne diseases. 

Although dengue is an important vector-borne disease, the literature regarding the costs and benefits of control options is relatively sparse [10]. Previous research has found that dengue fever poses a financial burden to households, not only in terms of the direct costs of dengue (e.g., hospitalization, treatment), but also indirect costs (e.g., loss of productivity, emotional stress) [11,12,13,14,15]. Studies have also evaluated the costs of malaria prevention at the household level [16,17,18] and documented the type and costs of mosquito control measures from malaria and dengue-endemic regions [19,20]. While these studies help to highlight the types and costs of products used in malaria endemic regions, few have gone further to examine a household’s reasons for buying mosquito control products [17,19]. Moreover, at the household level, protective measures to prevent Anopheles-transmitted malaria and *Ae. aegypti*-transmitted illnesses are different due to differences in vector behavior. For example, bed nets for night use are effective against Anopheles species, but largely ineffective against the day-biting *Ae. aegypti* mosquito. 

The aim of this study was to investigate the economic and socio-behavioral factors influencing household-level *Ae. aegypti* control in the city of Machala, a dengue-endemic area with high *Ae. aegypti* densities. This study was conducted in 2015, during the emergence of the chikungunya epidemic and one year prior to the emergence of Zika fever in the city. The study does not aim to address government costs of public health interventions (dengue prevention and control) or costs to the healthcare system for individual for cases of dengue fever. We aimed to assess the mosquito control products available in local markets, the costs incurred by households on dengue prevention and mosquito control, and the factors that influenced a household’s decision to purchase a mosquito control product. This evidence is intended to be an additional input for researchers and decision-makers examining the costs and feasibility of proposed vector control interventions.

## 2. Methods 

### 2.1. Study Site and Study Population

**Figure 1:**
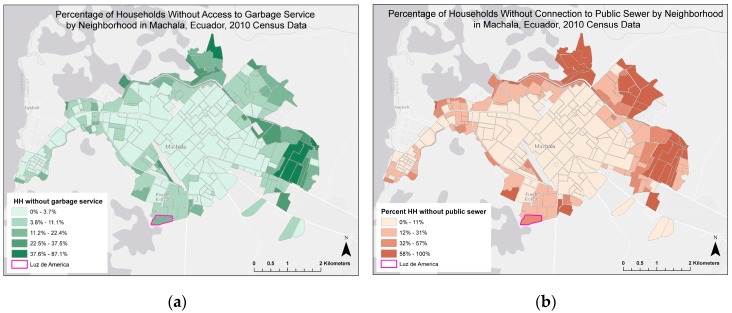
Location of study site in Machala, Ecuador. Luz de America indicated with neighborhood level census information from 2010 data: (**a**) Percentage of households without access to garbage service (21.2% for Luz de America) and (**b**) without connection to public sewerage system (37.7% for Luz de America). Data source: Instituto nacional de estadistica y censos (INEC). Neighborhood map source: National Institute of Meteorology and Hydrology of Ecuador (INAMHI).

Machala, Ecuador (population of approximately 246,000) is the capital city of El Oro Province and is a major port on the Pacific Coast, located 70 km north of the Peruvian border [21]. Machala is typical of mid-sized cities in Latin America that experienced rapid, unplanned growth from 1960 to 1980, resulting in uneven access to piped water, garbage collection, and paved roads in the urban periphery (Figure 1) [9]. We conducted this investigation in a low-income urban area located at the southernmost edge of the city, Luz de America (population of 1368, approximately 267 households). Population estimates were derived from vector control activities that transpired in the area from 2012 to 2013. The neighborhood was bordered by mixed commercial and residential buildings to the north and east, and mangroves and abandoned shrimp ponds to the southwest. Streets were unpaved and most households had access to sewerage, garbage collection, and piped water inside the home. Many households (43%) reported daily or weekly interruptions in the piped water supply, and thus, stored water in cisterns or elevated tanks (80%). 

From 2014 to 2016, during the peak dengue transmission season, the average *Ae. aegypti* Household Index (percentage of houses infested with larvae and/or pupae), was 14.78 and the average Breteau Index (number of positive containers per 100 houses inspected) was 21.40, per MoH records. Common *Ae. aegypti* larval habitat in this community included standing water in puddles, 55 gallon drums for water storage, tires, and discarded containers in and around the household patio. This neighborhood was selected because the MoH identified the community as a high-risk area for dengue, and because the MoH was not conducting any regular *Ae. aegypti* control interventions in the community during the study period.

This study was conducted from March 2015 to August 2015, in conjunction with a pilot field trial of a novel intervention to reduce the indoor density of *Ae. aegypti* populations. Forty households were selected based on (a) their willingness to participate in this study, and (b) that they did not own an air conditioning unit. 

### 2.2. Research Methods

The study was conducted in accordance with the Declaration of Helsinki and the protocol was reviewed and approved by the Institutional Review Boards at SUNY Upstate Medical University (project identification code: 2014-0), Syracuse University, University of Colorado, and the Central University of Ecuador. Heads of household (>18 years) were consented by trained field technicians, and signed an informed consent form prior to study start.

### 2.3. Product Surveys

To determine the types and costs of *Ae. aegypti* control products available for sale to the Luz de America community, we sampled 10 neighborhood stores (within one kilometer from the neighborhood limits) and three supermarkets, representative of the five large supermarkets in the city. Additionally, the primary outdoor market in central Machala was surveyed for items that could not be found at the other stores. At all stores, we documented the product name, size, active ingredient, availability, and price of the different mosquito control products. At the neighborhood stores and outdoor market, we asked store owners for the prices of products, whereas at the supermarkets, the prices listed on the tag below the products were documented. The products were categorized into repellents, aerosols, mosquito coils, liquid sprays, and other categories as needed. We took photos of the products and printed them on flashcards for use during the household surveys. 

### 2.4. Household Surveys

From March to June 2015 we surveyed the head of household or the responsible adult who resided in the home during the day in a face-to-face interview that solicited information about household demographics, dengue knowledge and perceptions, vector control and water storage practices, and barriers to employing prevention practices. This survey was a modification of an instrument that was used previously in a dengue surveillance study in Machala by project investigators [8,9].

Following the completion of the household surveys, we designed a semi-structured questionnaire to further explore the economic themes that emerged. We surveyed 38 of the same 40 households during the second half of the study period (July to August 2015). Two households were lost to follow up because the residents had moved. The information solicited included direct quantitative questions on household expenditures and income. For comparison, we consulted the latest data available on household income from Ecuador’s National Institute of Statistics and Census (INEC, by its Spanish acronym). As economic data specific to Machala was not available, we investigated national average wages from the most recent national survey in 2011–2012. Survey respondents were also asked open-ended questions regarding mosquito control and dengue prevention practices and the costs incurred. Using the flashcards from the store product surveys, survey respondents were asked to identify specific products purchased and the factors that influenced their decision to purchase the product. The survey instrument was piloted prior to study start. The survey instrument in English and Spanish can be found in Supplementary Materials.

Two trained local field workers conducted the interviews. The semi-structured questionnaire was audio recorded and later transcribed into Spanish text by project technicians. For the open-ended question regarding factors that influence the decision to purchase a product, we used standard qualitative theme analysis to identify emergent themes and the most common responses from the transcripts [22]. To estimate the relative importance of each response, we created a database of codes in excel, and tabulated the number of households from which each response emerged. Coding in Spanish was manually conducted by NH and cross-validated by AMSI.

### 2.5. Analysis

A priori, we identified five variables (home ownership, prior chikungunya or dengue infection, stability of job, household income below minimum wage, gender) that we thought would influence mosquito control expenditures and ran independent sample *t*-tests to evaluate the evidence that the associated population means were significantly different. We also compared total number of strategies employed to expenditures on mosquito control using Pearson parametric correlation tests. Finally, we categorized number of mosquito control strategies employed by households into high/moderate or low and compared the means using a two-sample *t*-test in the two groups to the response no difficulties (1 = responded “no difficulties”, 0 = other response) to mosquito control. All analysis was done in R version 3.3.1 [23]. 

## 3. Results

### 3.1. Household Demographics

Thirty percent (*n* = 12) of the heads of households were female and a majority had either completed primary (43%) or secondary (55%) education (Table 1). One-quarter of respondents (*n* = 10) rented their home. The median number of persons living per household was five. Many of the households had access to municipal sewerage (83%) and piped water inside the home (65%). However, 43% (*n* = 17) reported daily or weekly interruptions in the piped water supply. Eighty percent (*n* = 32) of households store water in cisterns or elevated tanks. The water stored is non-drinking water used for cooking, washing dishes/clothes, showering, cleaning the home, etc. 

In the community where we conducted our studies, median weekly family income was US$107.50 (range: US$50.00, 250.00). Eighty-five percent (*n* = 34) of the heads of households are employed, however 38% (*n* = 13) of this employed group have a job that is unstable and 41% (*n* = 14) reported that the head of household earns less than the minimum wage, US$350 per month. In comparison, the average national monthly income for households in Ecuador was US$709.

#### Knowledge and Perceptions of Vector Control

Most survey respondents (88%) reported that dengue was a serious problem in their communities (Table 1). Approximately two-thirds reported that someone in their family had been ill from dengue or chikungunya, and through open-ended responses, they identified dengue as one of the top three health concerns in their community. Ninety percent were aware that dengue was transmitted by mosquitoes. Economic limitations were identified by survey respondents as the most common challenge to employing vector control strategies, reported by 38% of respondents, followed by no difficulties (32%) as the second most common answer. In our statistical analysis, we found that the mean in the high/moderate utilizers of mosquito control activities that reported no difficulties (M = 0.167) was significantly lower than the mean in the low utilizer group (M = 0.456) with a *p*-value < 0.05. 

### 3.2. Household Mosquito Control and Dengue Prevention Interventions

Survey respondents in the semi-structured survey reported employing a median of five different mosquito control and dengue prevention strategies in their household (range: 0, 10) including chemical control, elimination of larval habitat, mosquito avoidance behaviors, and mosquito repellent strategies (Table 2) when asked the open-ended question, “what strategies do you use to prevent dengue transmission in your home and reduce the number of mosquito bites?”. The most commonly reported mosquito-control practice was the use of bed-nets, however, almost all respondents reported only using bed-nets at night. Likewise, households reported closing doors and windows to prevent mosquitoes from entering the home only in the evening hours. 

### 3.3. Store Product Surveys

Surveys of small neighborhood stores showed that all 10 stores sold at least one type of mosquito control product, defined here as any product that kills or repels mosquitoes. Five different mosquito control products were found; the most common product was Dragon (active ingredient: tetramethrin) liquid insecticide 450 mL (median price: US$2.50). The three large supermarkets had far more variety and availability of mosquito control products (Table 3). The most abundant types of product available were liquid insecticides and mosquito coils. The only items used by households and not found in neighborhood stores or supermarkets were bed-nets and Palosanto (*Bursera graveolens*), a type of wood burned to create smoke, which acts as a spatial repellent against mosquitos. These two items were only available at the central outdoor markets. The price of an untreated bed net ranged from US$9.00 to US$35.00 (median: US$13.50) depending on material and size. The price of Palosanto was about US$1.00 for a small bag, which contained about five small sticks. 

### 3.4. Household Expenditures

We found that households typically spent US$2.00 on mosquito control per week representing 1.90% (range: 0.00%, 9.21%) of median weekly income (Table 4). After accounting for food expenditures, the average household was left with US$27.50 weekly (US$3.93 daily), for use for housing, water, electricity, transport, communication, recreation, health, etc.

### 3.5. Factors Influencing Decision to Purchase a Product

From the analysis of coded open-ended responses, we found that the effectiveness of a product, low cost, and ease of use/application were the most important factors that influence people’s decisions to purchase a mosquito control product (Table 5). Other important factors, reported by one-third of households, included minimal health effects and a recommendation by a friend or family member. For effectiveness, respondents determined the success of a product either by seeing dead mosquitos on the ground or experiencing fewer mosquito bites. Several respondents reported purchasing a product because a friend/family member has recommended the product or out of curiosity, but they only continued to purchase the product if they experienced good results. The following quotes are representative of responses to “what factors influence your decision to purchase a mosquito control product?”

Example Quote 1. “*I am not accustomed to buying the same item. If I see something new I like to try it to see how it works. If it is efficient and works then I will buy it again.*”

Example Quote 2. “*I buy incense (mosquito coils) because it was effective for my mom…the moment it is turned on the mosquitos disappear. It is very effective.*”

The second and third most important factors on whether to purchase a product were the cost and ease of use/application, respectively. Whereas low cost of a product saves money, ease of use of a product saves time. Some liquid spray insecticides are sold with the spray nozzle attached to be used immediately; another option is to buy the fumigation pump, a method used by households that mix diesel with liquid insecticide.

Health concerns, or minimal effects on health, were identified as an important factor by 29% of households. Some of the perceived harmful effects were allergies, breathing problems, irritation of the throat, and harm to the lungs. Products that generated smoke and aerosolized insecticides were identified as the strategies that households had most frequently discontinued. Health concerns were the predominant reasons for stopping these practices. Several households reported that, even though they understood the health concerns associated with use of insecticides, they continued to fumigate, but changed their fumigation practices. For example, respondents would fumigate and leave the home for 10-15 minutes and/or wait to do so when children were at school.

Example Quote 3. “*After I fumigate, I leave the home. I know not to enter. If I do, I will fall, just like the mosquitos.*”

### 3.6. Predictors of Monthly Expenditures

The strongest predictor of mosquito control and dengue prevention expenditures was whether the occupants of the household owned (Mean = US$9.90) or rented (M = US$5.11) their home (*p* = 0.058, Table 6). We found a slight increase in expenditures in households that self-reported that someone in the family had fallen ill from dengue or chikungunya (M = US$9.71) compared to households with no prior infections (M = US$8.14), although the difference was not statistically significant (*p* = 0.590). We found that households where the head of household had a stable job spent more on mosquito control and dengue prevention (M = US$9.71) than those with an unstable job (M = US$7.44), although the difference was not statistically significant (*p* = 0.406). We found no association between monthly mosquito control and dengue prevention expenditures and household income above/below minimum wage (*p* = 0.872) and gender of the head of the household (*p* = 0.884). In correlation analysis, we found a moderate positive association between total number of strategies employed and expenditures on mosquito control and dengue prevention strategies (*p* = 0.000). 

## 4. Discussion

As countries throughout the tropics and subtropics face the rising epidemics of *Ae. aegypti*-transmitted febrile illnesses—such as dengue, chikungunya, and Zika fever—socio-economic research on *Ae. aegypti* control is urgently needed to inform the development and implementation of novel mosquito control strategies. Novel *Ae. aegypti* control technologies are promising (e.g., sterile insect methods via Wolbachia infected mosquitoes and genetically modified mosquitoes) [24,25,26]. However, the effectiveness of their implementation depends largely on community perceptions of risk, perceptions of the relative effectiveness of these strategies, and the economic and behavioral barriers to implementation at the household-level [9,27,28,29,30,31,32].

The burden of *Ae. aegypti* febrile illness is a major public health priority in Machala, as reflected by community perceptions of risk and prevalence. The results of this study indicate that households in low-income communities spend more than 10% of their family discretionary income on interventions related to mosquito-borne disease. Discretionary income, or income that is left for spending after paying for household necessities (e.g., food and shelter) was calculated based on national results from INEC data. A typical household in Ecuador spends about 8% of household income on housing, water, electricity, gas, and other fuels. In Luz de America, a family with the median weekly income (US$107.50) can be expected to spend about US$8.50 on housing, water, electricity and gas. After accounting for median self-reported expenditures on food (US$80.00) households are left with US$19.00 weekly on discretionary income. Of this amount, the US$2.00 on mosquito control and dengue prevention strategies represents more than 10% of discretionary income. 

Households in this study employed five mosquito control interventions to reduce the burden of *Ae. aegypti* transmitted illness. Our findings are consistent with a previous study in Machala both in terms of the number and diversity of strategies employed by households to prevent dengue fever [9]. However, there is little empirical evidence that these interventions effectively prevent disease. Indeed, a majority of these households self-reported that someone in their family had been ill with dengue or chikungunya. Novel interventions that are both low cost and effective at reducing the population of *Ae. aegypti* are urgently needed. This study can help guide the development of novel mosquito control strategies for robust markets, such as Ecuador, and help governments decide how to spend on vector control interventions, by comparing the economic burden to households to the cost of other type of interventions [33]. Innovative control strategies are promising, such as lethal oviposition traps, use of transgenic *Ae. aegypti* that prevents larvae from developing into adulthood, and attractive toxic sugar baits [34,35,36,37]. The success of these strategies will rest on common themes of economic and behavioral barriers that can impede or assist in implementation at the household-level.

We found that economic limitations were the most important barrier to household-level vector control followed by the response “no difficulties.” Home ownership and stability of the job of the head of the household, proxies of poverty, were associated with expenditures on mosquito control and dengue prevention. Our findings support reports by prior studies on malaria prevention, which found that prior to the mass distribution of free insecticide-treated mosquito nets, that achieved great equity in coverage, access to bed nets was largely dependent upon wealth [17,38,39,40]. In low-income communities, financial subsidies for mosquito control interventions will be a key strategy to promote use. Households that reported no challenges to vector control were more likely to employ fewer strategies to vector control. Combining our narrative, we interpret this to mean that the ease of access in the neighborhoods, variety of products available, and the low cost of certain products may make it very easy, and with no reported difficulties, to carry out mosquito control strategies in the household, especially in households that report employing very few mosquito control strategies.

We identified key factors that influence people’s decisions to purchase a mosquito control product, with important implications for interventions. For effective *Ae. aegpyti* control to take place in this area, interventions must be simultaneously effective, low cost, and easy to use. In our study, many respondents had stopped using insecticide sprays and products that produce smoke because of health concerns. While supermarkets sold a range of products (repellents, aerosols, mosquito coils, liquid sprays, and equipment), the easiest to find in neighborhood stores were tetramethrin-based products. Cypermethrin and tetramethrin, active ingredients used in popular insecticide sprays, are classified as possible human carcinogens [41,42]. Prolonged exposure to smoke from mosquito coils may have harmful effects on the lungs or mutagenic effects [43,44]. Families have to make difficult decisions, balancing the health effects of certain products against the health effects of diseases transmitted by *Aedes* mosquitoes. 

The survey results indicate that the community knowledge was very high on the transmission of dengue, and community perceptions were very high regarding the severity, importance, and risk of dengue fever. Focus groups conducted in Puerto Rico found that participants with a previous dengue diagnosis were more concerned about risk of the disease and recommended using repellent more often than their counterparts without a previous dengue diagnosis [45]. Additional studies in Thailand and Malaysia have documented a direct link between knowledge on dengue prevention and prevention practices [46,47]. More specific questions on community knowledge of dengue epidemiology and vector bionomics would be a primary interest in Machala because of an increasing trend of *Ae. aegypti*-transmitted illnesses in this highly populated urban environment. In South America, continued evaluation of people’s knowledge, perceptions, and practice are of great importance to improve integrated control measures and organize health education programs to resolve the dengue problem.

This study provides an important perspective on *Ae. aegypti* control, despite the small sample size of the study population. We chose our study site in Machala because this was an area where the MoH had stopped the systematic fumigation and larviciding of homes due to resource limitations. This community was typical of low-income communities in the urban periphery in coastal Ecuador, where the political-institutional and community-household determinants that result in high densities of *Ae. aegypti* are intimately connected with poverty. For example, risk factors such as inadequate access to municipal public services and utilities (e.g., garbage collection, sewerage, piped water) are prevalent in economically disadvantaged areas and can lead to an increase in *Ae. aegypti* densities. Previous work in Machala found that residents in the urban periphery felt neglected by government institutions [9]. In our study population, residents have taken it upon themselves to prevent dengue fever and other mosquito-borne diseases in their family. However, for most of the residents, mosquito control expenditures represented a substantial proportion of their income, and individual household protection may not be effective at preventing disease. Innovative vector control interventions that focus on community mobilization can yield more cost-effective solutions [29,33,48]. Community-based pilot dengue control programs by the MoH in Machala have been found to effectively reduce densities of *Ae. aegypti* [49,50,51]; however, the sustainability and scalability of these interventions has been challenged due to the recent restructuring of the MoH and elimination of the national vector control program.

A limitation of this study was determining the period during which people used the different mosquito control products. Future studies should include a specific time period when asking, “which of these products do you purchase?” We chose not to report the percentage of household’s responses to that question because recall periods could bias the results. For smaller consumption items, appropriate recall periods are typically two and four weeks in economic household surveys [52]. In this study, surveys were conducted at one time point in one community. Future studies would ideally repeat the questionnaires over a period of one year, to capture seasonal variation in consumer behavior, and in a range of communities representing different income levels. Since our interviews took place at the end of the rainy season, the responses by study participants may underestimate true expenditures during the peak of dengue transmission. 

Previous dengue or chikungunya infection was self-reported by respondents. We did not differentiate between dengue or chikungunya affected households. With the rise of chikungunya and Zika throughout Latin America, the distinction between *Ae. aegypti-*transmitted illnesses is an important step in order to measure and compare the perception of risk for each of the diseases and attribute the adoption of vector control strategies to the respective disease. It is well-known that acceptable risk is lower for unknown and unfamiliar activities [53]. Zika’s newness and unfamiliarity may have important implications for perception of risk and the adoption of preventative strategies.

A strength of this study was the validation of product prices from different sources. The prices self-reported by residents were found to be very similar to prices documented during the product surveys. These results provide important information about the types of products used by households and relative amounts spent per product. 

## 5. Conclusions

The results of this study demonstrate that households in the urban periphery spend a considerable proportion of discretionary household income to prevent mosquito-borne diseases. These findings also show a robust and healthy market for commercial mosquito control products, even among the poorest of households in Machala, Ecuador. 

Dengue has a high economic impact, but the true economic cost of the disease is unknown. A 2015 literature review from the Costing Dengue Working Group in the Americas found only a few economic studies in the region [54]. The published literature is lacking in quantity and quality. The cost studies are many times unclear and not representative of the total economic costs of dengue, particularly the prevention costs to households [55]. The importance of determining the economic burden of dengue prevention at the household level is so that it can be used for cost-effectiveness analyses to provide important information about efficient resource allocation [56].

This study addresses the need for economic research specific to dengue prevention at the household level to ensure informed decision making on the various options for controlling and preventing *Ae. aegypti* transmitted diseases. The findings from this study contribute to a body of research on the types and costs of interventions used by households that face a high burden of *Ae. aegypti*-transmitted illnesses, such as dengue, chikungunya, and Zika. We also identified the barriers and factors that influence a household’s decision to purchase a mosquito control product. This information is important to understand the role of these interventions in prevention of mosquito-borne diseases and economic burden imposed on households.

Helping community members to protect themselves against dengue fever and other vector borne diseases is a critical public health objective. Interventions that are low cost, effective, easy to use, and pose minimal health risks to families will have the greatest chance of widespread use. Even with vaccines available in the future, vector control is likely to remain a key means of prevention of *Ae. aegypti*-transmitted illnesses. With the rise of chikungunya and Zika fever, more work must be done to identify interventions that efficiently reduce *Ae. aegypti* densities, especially in vulnerable communities that face the greatest social and economic burden.

## Figures and Tables

**Table 1:** Socio-demographic information from survey respondents (*n* = 40).

Socio-Demographics	% Households (*n*)
Female head of household	30% (12)
House is rented	25% (10)
Head of household has only primary education	43% (17)
Head of household has secondary education	55% (22)
Head of household is currently employed	85% (34)
Employed and earns less than the minimum wage (*n* = 34)	41% (14)
Employed and job is unstable (*n* = 34)	38% (13)
Water access & storage	
Access to municipal sewerage	83% (33)
Piped water inside the house	65% (26)
Daily or weekly interruptions in the piped water supply	43% (17)
Water stored in cisterns or elevated tanks	80% (32)
Housing Condition	
Have screens on all windows and doors	20% (8)
Patio condition is good (*n* = 29)	17% (5)
General housing condition is good	15% (6)
Knowledge & perceptions	
Someone in the family with prior dengue or chikungunya infection	60% (24)
Knowledge that dengue is transmitted by mosquitoes	90% (36)
Consider dengue to be a serious problem in the community	88% (35)
Dengue is one of the three most important health problems in the community (open-ended response).	65% (26)
Primary challenge to vector control (only one response selected)	
Economic limitations	38% (15)
Lack of information	10% (4)
Lack of time	20% (8)
No difficulties	32% (13)

**Table 2:** Mosquito control and dengue prevention strategies reported by survey respondents (*n* = 38).

Mosquito Control and Dengue Prevention Strategies	% Households (*n*)
Sleep under bed-net	92% (35)
Close windows and doors	61% (23)
Cover tanks with water/do not let water accumulate outside	55%(21)
Fumigation	53% (20)
Burn plants for smoke	50% (20)
Eliminate trash	50% (19)
General cleaning	45% (17)
Apply repellent	32% (12)
Use liquid larvicide provided by the MoH *	29% (11)
Apply diesel to floors	26% (10)
Cut vegetation	24% (9)
Use other insecticides	24% (9)

* *Bacillus thuringiensis* subspecies *israelensis* (*Bti*) was being used by the MoH (Ministry of Health) at the time of the study. *Bti* contains spores that produce toxins that specifically target the larvae of the mosquito. When resources are adequate, the MoH administers liquid larvicides at no cost to the household.

**Table 3:** Mosquito control and dengue prevention products available in markets in Machala in 2015.

Type of Product	Product Brand	Description (Available Products)	Main Active Ingredient	Median Price per Unit or per mL ($US)	Price Range per Item ($US)
Repellent	Detan	Lotion 60 mL, 120 mL	Diethyl Toluamide “deet”	0.02	2.09–2.99
	OFF!	Spray 127 mL	Diethyl Toluamide “deet”	0.05	6.59–6.59
Liquid insecticides	Dragon	Liquid 230 mL, 450 mL, 950 mL, 475 mL w/spray nozzle	Tetramethrin 0.46%	0.01	1.34–4.39
	Torvi	Liquid 230 mL, 500 mL, 1000 mL, 630 mL w/spray nozzle	Cypermethrin 0.25%	0.01	1.26–4.05
	Flit-Kit	750 mL w/ spray nozzle	Cypermethrin	0.01	3.49–3.49
Mosquito coils	Incienso	10 spiral units	D-Allethrin 0.20%	0.10	0.99–0.99
	Baygon	6 double spiral units	D-Allethrin 0.20%	0.18	1.00–1.19
	Aguila	10 spiral units	Dimefluthrin 0.02%	0.10	0.90–0.98
	Lanju	10 spiral units	Dimefluthrin 0.03%	0.09	0.90–0.95
Aerosol sprays	Sapolio	Spray 235 mL	D-Tetramethrin 0.15%	0.01	3.00–3.85
	Raid	Spray 235 mL, 360 mL	D-Tetramethrin 0.35%	0.01	3.20–4.81
	PIX	Spray 300 mL	Chlorpyrifos 0.5%	0.01	3.03–3.03
	Rodasol	Spray 400 mL	Bioallethrin 0.2%	0.01	4.24–4.39
Equipment	Mosquito Racket	Rechargable racket		4.49	3.99–4.99
	Dragon	Insecticide pump		2.69	2.39–2.99
	(Bed-net)	Untreated bed-net, varying sizes and styles		13.50	9.00–35.00
Other	Palosanto	Small bag of five sticks		0.20	0.75–1.25

**Table 4:** Weekly household expenditures reported by survey respondents (*n* = 38).

Measures	Median (US$)	Min (US$)	Max (US$)	% of Weekly Income
Income	107.50	50.00	250.00	100.0%
Food expenditures	80.00	30.00	120.00	74.0%
Mosquito control expenditures	2.00	0.00	9.21	1.9%

**Table 5:** Summary of open-ended responses regarding factors that influence the decision to purchase a mosquito-control product (*n* = 38).

Factors	% Households (*n*)
Effective product	42% (16)
Low cost	39% (15)
Easy to use/apply	34% (13)
Minimal effects on health	29% (11)
Recommended by a friend/family member	29% (11)
Easy access in my neighborhood	21% (8)
Accustomed to use/always have used the product	11% (4)
Heard about the product on TV/news/radio	8% (3)
Product kills mosquitoes (vs repel)	8% (3)
The product lasts for a long time	8% (3)
Co-benefits of use, i.e., product kills other pests also	5% (2)

**Table 6:** Bi-variate associates of hypothesized predictors of mosquito control and dengue prevention expenditures.

Measure	*n*	Mean (US$)	*p*-Value
Home ownership	40	Own = 9.90 Rent = 5.11	0.058 ^1^
Someone in the household with prior chikungunya or dengue infection	38	Yes = 9.71 No = 8.14	0.590
Stability of job	40	Stable = 9.71 Unstable = 7.44	0.406
Household income above/below minimum wage	38	Above = 9.00 Below = 10.86	0.872
Gender	40	Male = 8.56 Female = 9.00	0.884
Total strategies employed ^2^	40	*r* = 0.571	0.000 ^1^

^1^ Significant at *p* = 0.1 level; ^2^ Pearson parametric correlation test.

## Supplementary Materials

The following are available online at www.mdpi.com/1660-4601/14/2/196/s1, Survey instrument in English and Spanish.
