# Supplementary material for: Household Dengue Prevention Interventions, Expenditures, and Barriers to Aedes aegypti Control in Machala, Ecuador
Source: Int J Environ Res Public Health. 2017 Feb 16;14(2):196. doi: 10.3390/ijerph14020196 (PMC5334750; doi:10.3390/ijerph14020196)
Supplement: Supplementary file 1 [file ijerph-14-00196-s001.pdf]

Supplementary Materials: Household Dengue Prevention Interventions, Expenditures, and Barriers to *Aedes aegypti* Control in Machala, Ecuador

Naveed Heydari, David A Larsen, Marco Neira, Efraín Beltrán Ayala, Prissila Fernandez, Jefferson Adrian, Rosemary Rochford and Anna M Stewart-Ibarra

Supplement : Survey Instrument (English and Spanish Versions)

HOUSEHOLD SURVEY

Household Identification Number___________ Date (dd/mm/yy):__________________

Interviewer name:

____

INFORMATION ABOUT THE HEAD OF THE HOUSEHOLD

Age:_______ years Sex: ☐ Male ☐ Female

Does this person work? ☐ Yes ☐ No: Specify why not (e.g., retired, looking for work, disabled)_________________

If they do work, is this stable employment? ☐ Yes ☐ No

Do they earn the minimum wage? ☐ Yes ☐ Less than the minimum wage ☐ More than the minimum wage

What is their highest level of education? ☐ None ☐ Primary ☐ Secondary ☐ Post-secondary

HOUSEHOLD DEMOGRAPHICS

How many people sleep in this household?_______ How many families sleep on this property?____________

Do you rent this house or do you own the home? ☐ Rent ☐ Own

Are there other families that rent on this property? ☐ Yes ☐ No

---

ACCESS TO BASIC SERVICES AND WATER USE

The water that this household receives is:

☐ piped water inside the home ☐ piped water outside the home ☐ No piped water

When you open the faucet (either outside or inside the home) how often is there NO water?

☐ there is always water ☐ daily ☐ 2-3 times per week ☐ other

The household sewerage system is: ☐ municipal sewerage ☐ septic tank ☐ other___________

How often is garbage collected in your neighborhood?

☐ Never ☐ Daily ☐ 2-3 times per week ☐ Other:________

What do you use to ventilate the household? ☐ AC ☐ open the door/window ☐ fan

Do you have a cistern or elevated water tank that is in use? ☐ Yes ☐ No

Do you store water apart from the water in the cistern or elevated water tank? ☐ Yes always ☐ Yes sometimes ☐ No

IF YES, Why do you store water? ☐ There is no piped water in my home

☐ There are frequent interruptions in the water supply ☐ It is convenient/habit ☐ Otro:_____________

For what do you use the stored water? ☐ laundry ☐ clean the house☐ drinking water ☐ cooking ☐ bathing ☐ to water the plants

Do you have animals? ☐ dogs ☐ cats ☐ chickens/ducks ☐ pigs ☐ birds ☐ Otros:_________

PERCEPTIONS AND KNOWLEDGE OF DENGUE

In your opinion, what are the three most serious health problems in your neighborhood?

Do you consider dengue to be a problem in your community?

☐ Yes it is a serious problem ☐ Yes it is a problem, but not so serious ☐ No its not a problem

How is dengue transmitted? ☐ mosquito ☐ other:________________

PREVENTION ACTIONS

Do you do any of the following actions to prevent dengue?

- ☐ apply chemicals to standing water
- ☐apply repellent
- ☐ burn palosanto
- ☐ pour burned diesel on the floors/puddles
- ☐ Fumigate my house
- ☐ Use mosquito net

How much do you spend per month on these mosquito control interventions? $________

What are the principal limitations to taking these prevention actions (and why)? ☐ No difficulties

☐ lack of information:__________________

☐ economic limitations:________________

☐ lack of time:________________________

☐ other:_______________

CHARACTERISTICS OF THE HOUSEHOLD: OBSERVATIONS OF THE INVESTIGATOR

---

1. The overall condition of the house is

☐ Good (new, well maintained) ☐ Regular ☐ Bad (old, unpainted, uncared for)

2. The material of the external walls of the house?

☐ brick/concrete blocks ☐ wood ☐ bamboo ☐ other:_____________

3. How many bedrooms in the home? ______

4. Do doors and/or windows have screens? ☐ Yes, all ☐ Yes, some ☐ No, none

5. What is the condition of the screens? ☐ No screens ☐ good (new) ☐ normal ☐ bad (old, holes)

6. Principal access to the household: ☐ Paved street ☐ dirt road ☐ Other:__________

7. Are there abandoned properties or homes adjacent? ☐ yes ☐ No

8. Is there a patio? ☐ yes ☐ No

10. Condition of the patio:

☐ Very organized/clean (no garbage, garden well maintained) ☐ Normal(little garbage) ☐ Disorganized

11. Shade in the patio: ☐ Sunny (<25% shaded) ☐ Partial (25%-50%) ☐ Shady (>50% shaded)

HOUSEHOLD EXPENDITURES/MOSQUITO ABATEMENT EXPENDITURES

[To the study participant]: In the next section we will ask you a few questions about your household expenses. All information is strictly confidential. We want to understand more about the cost of dengue control in the household, since this is an important factor that limits people's ability to prevent the disease. Also, we want to understand how much families in Machala would be willing to spend to prevent dengue, as new ways to control mosquitoes became available.

(The interviewer should not force consistency between the totals reported in one question and the sum of answers in the following ones. All amounts should be recorded in local currency.)

BACKGROUND/CONTEXT:

Has anyone in your family been ill with dengue or chikungunya?

If yes, how recently?

HOUSEHOLD DENGUE PREVENTION STRATEGIES

What strategies do you use to prevent dengue transmission in your home and reduce the number of mosquito bites? (don't read options at first, allow for free brainstorming, then prompt with the options that have not been mentioned)

Intervention	Frequency: rainy season (peak dengue)	Frequency: now (low dengue season)	Who in the household is responsible?	How much time does it take per day or per week?	Costs?
Replace screens					
Eliminate garbage					
Cut vegetation					
Apply repellant					
Burn palo santo or other grass to make smoke					
Use bactivec (liquid larvicide given by the MoH) or granular larvicide (abate)					
Use other pesticides inside the house					
Shut windows and doors					
Sleep under mosquito nets when ill					
Burn diesel					

Are there any dengue prevention methods that you used in the past, but no longer continue to use?

If yes, why did you stop using those methods?

In order to understand the relative cost of mosquito abatement in your household, I will ask a few questions about your other household expenditures.

In one week, approximately how much does your household spend on food (Food expenditures

include all food prepared and consumed by the household, along with food purchased and consumed outside the household (e.g. snacks, bag lunches). However, should exclude meals at sit-down restaurants, and expenditures on alcohol and tobacco.)

Approximately how much does your family earn each week?

WILLINGNESS TO PAY

How much would you be willing to spend per week to completely eliminate the dengue mosquitoes from your household?

The purpose of the 16-week research study when we collected mosquitos is to explore new methods of killing mosquitos to control dengue, specifically the mosquito killer we asked you to hang inside your house for 4 weeks. (Show photo of ATSB device). As a reminder, the mosquito trap is filled with sugar water and a minimal amount of boric acid.

How much would you pay for this specific device, assuming it lasted for 1 month and you needed 1 device per room of your home?

ANTI-MOSQUITO PRODUCTS

Here are six flashcards with pictures of anti-mosquito products that you can find in your local shops. These products can potentially reduce the risk of dengue in your household. (Show each flashcard one by one and read the short description).

Which of these products do you purchase? (Write down corresponding letter)	How much do you spend?	How often do you use these products?	Prompt Question: What factors influence whether you choose a product? ((don't read options at first, allow for free brainstorming, then prompt with the options that have not been mentioned)

Options for prompting question above: (A) it was recommended by a family/friend, (B) low cost, (C) minimal health effects, e.g., respiratory issues due to chemicals, (D) easily accessible in my neighborhood, (E) I have used the product in the past and know that it is effective, (F) easy to apply/use, (G) it is a new product that I heard about on the news/radio/tv and I want to try it out, (H) the Ministry of Health has recommended the product

ENCUESTA DEL HOGAR

Número de Identificación de la casa

(Agregar código aquí)

____

Fecha (dd/mm/aa):_________________

Nombre del entrevistador:

INFORMACION SOBRE EL/LA PRINCIPAL RESPONSABLE ECONÓMICO DEL MANTENIMIENTO DE SU FAMILIA

Edad: _________ Años Género: ☐ Masculino ☐ Femenino

Trabaja? ☐ Sí ☐ No: Especifique por qué no (ej., jubilado, estudiante)_____________________________________

Si trabaja: Su trabajo es estable? ☐ Sí ☐ No

Gana el salario básico? ☐ Sí ☐ No

Cual es el nivel de educación más alto al que esa persona asistió ?

☐ Ninguno ☐ Primario ☐ Secudario ☐ Post-secundario

EL HOGAR

Cuántas personas en total duermen en su hogar?_______ Cuántas familias duermen en esta propiedad?__________

Alquila su vivienda o es casa propia? ☐ Alquilo ☐ Casa propia

Hay otras familias que alquilen esta vivienda? ☐ Sí ☐ No

ACESO A SERVICIOS BÁSICOS Y USO DE AGUA

El agua que recibe la vivienda es:

☐ por tubería dentro de la vivienda ☐ por tubería fuera de la vivienda ☐ No recibe agua por tubería

Cuando abres la llave (adentro o fuera de la casa) con qué frecuencia no cae el agua?

☐ Siempre cae el agua ☐ Interrupciones diarias o semanales ☐ Otro:______________

El servicio higiénico o escusado de la vivienda es por: ☐ Alcantarillado ☐ Pozo séptico ☐ Otro:__________

Cada cuánto tiempo recogen la basura en su barrio?

☐ Nunca ☐ Diario ☐ 2-3 veces a la semana ☐ Otro:________

Qué utiliza para ventilar/refrescar la casa? ☐ Aire acondicionado ☐ Abrir ventana/puerta ☐ Ventilador

Tiene usted una cisterna o tanque elevado que está en uso? ☐ Sí ☐ No

Almacena usted agua aparte de en la cisterna o tanque elevado? ☐ Sí, todo el tiempo ☐ Si, a veces ☐ No

SI ALMACENA AGUA: Por qué almacena agua (*lea las opciones y marque todos las que apliquen*)?

☐ No hay agua entubada adentro de la casa ☐ El agua se corta con frecuencia

☐ Es conveniente / es costumbre ☐ Otro:_____________

Para qué se utiliza el agua almacenada (*lea las opciones*)? ☐ Para lavar ☐ Para limpiar la casa ☐ Para tomar (consumo humano) ☐ Para concinar ☐ Para bañar ☐ Para regar a las plantas

Tienen animales (*lea las opciones*)? ☐ perros ☐ gatos ☐ pollos/patos ☐ chanchos ☐ pájaros ☐ Otros:_________

PERCEPCIONES Y CONOCIMIENTO SOBRE DENGUE

En su opinión, cuales son las 3 principales problemas de salud que se han presentado en su barrio?

En su opinión, considera que el dengue es un problema en su comunidad? (*Lea todas las opciones*)

☐ Sí, es un problema muy serio ☐ Sí, es un problema, pero no tan serio ☐ No es un problema

Cómo se transmite el dengue de una persona a otra? (no lea las opciones):

☐ mosquito/mosco ☐ otro:________________

MEDIDAS DE PREVENCION

Cuáles de las siguientes medidas realizas para prevenir el dengue en su hogar? (*Lea todas las opciones*)

☐ Agrega quimicos en el agua para matar la larva

☐ Regar diesel quemado en los pozos o en el piso

☐Aplicarme repelente.

☐ Fumigación (rociamiento) al interior de la casa

☐ Quemar Palosanto o rollos chinos. ☐ Usar toldo/malla contra mosquitos

Cuanto es el gasto mensual por realizar estas medidas de prevención? $____________

Cuáles son las principales limitaciones para llevar a cabo estas medidas de prevención (y porque)?

☐ No hay dificultades

☐ falta de informacion :______________________

☐ falta economico :________________________

☐ falta de tiempo :________________________

☐ Otros:________________________________

CARACTERISTICAS DE LA VIVIENDA: OBSERVACIONES DEL ENCUESTADOR

1. El estado en general de la casa es:

☐ Bueno (nueva, bien mantenida) ☐ Regular ☐ Malo (vieja, despintada, descuidada)

2. El material predominante de las paredes exteriores de la vivienda?

☐ Ladrillo o bloque ☐ Madera ☐ Caña revestida o bahareque ☐ Otro:_____________

3. Cuántas habitaciones para dormir tiene la vivienda? ______

4. Las ventanas y/o puertas tienen mallas? ☐ Sí, todas ☐ Si, algunas ☐ No, ninguna

5. Cuál es el estado de las mallas? ☐ No hay mallas

☐ Bueno (nuevas, bien mantenidas) ☐ Regular ☐ Malo (viejas, con agujeros)

6. Acceso principal a la vivienda: ☐ Calle pavimentada ☐ Calle de tierra ☐ Otro:________________

7. Hay viviendas deshabitadas o terrenos baldíos alrededor de la vivienda? ☐ Sí ☐ No

8. Hay un patio trasero? ☐ Sí ☐ No

9. Condición del patio:

☐ Muy organizado/limpio (sin basura, jardín mantenido) ☐ Medianamente organizado (poca basura) ☐ Desorganizado

10. Nivel de sombra en el patio: ☐ Soleado (<25% sombra) ☐ Parcial (25%-50%) ☐ Sombreado (>50% sombra)

GASTOS DE LOS HOGARES/GASTOS DE CONTROL DE MOSQUITOS

[Al participante del estudio]: En la siguiente sección nosotros haremos unas preguntas acerca de los gastos de su hogar. Toda la información es estrictamente confidencial. Queremos entender más sobre el costo para el control del dengue en su hogar, debido que este es un factor importante que limita la capacidad de las personas para prevenir esta enfermedad. También, queremos entender cuanto están dispuestos a gastar las familias en Machala para prevenir el dengue, ya que nuevas formas de controlar los mosquitos llegaron a estar disponibles.

(El entrevistador no debe forzar la coherencia entre los totales reportados en una pregunta y la suma de las respuestas en las siguientes. Todas las cantidades deben ser registradas en la moneda local.)

HISTORIAL/CONTEXTO

Ha tenido alguien en su familia dengue o chikingunya?

Si responden que sí, hace cuánto tiempo?

ESTRATEGIAS DE PREVENCIÓN DEL DENGUE EN EL HOGAR

Que estrategias utiliza para prevenir la transmisión del dengue en su hogar y reducir el número de picaduras de mosquitos? (No leer las opciones al principio, permitir la lluvia de ideas libre, entonces sugerir las opciones que no se han mencionado)

Intervención	Frecuencia: estación lluviosa (pico de dengue)	Frecuencia: ahora (temporada de dengue bajo)	Quién es el responsable en el hogar?	Qué tiempo se tarda por día o semana?	Costos?
Remplazar mallas de ventana					
Eliminar basura					
Cortar vegetación					
Aplicar repelente					
Quemar palo santo u otras hierbas para hacer humo					
Usa bactivec (larvicida liquido entregado por MSP) u larvicida granulado (abate)					

Usa otros pesticidas dentro su hogar					
Cierra ventanas y puertas					
Duerme bajo toldo cuando está enfermo					
Aplica diésel					

Hay algunos métodos de prevención de dengue que tú usaste en el pasado, pero que ya no continúas usando?

Para entender el costo relativo de control de mosquitos en su casa, voy a hacer algunas preguntas acerca de sus otros gastos del hogar.

En una semana, aproximadamente cuánto gasta en comida en su hogar? (Los gastos de alimentos incluyen todos los alimentos preparados y consumidos por el hogar, junto con los alimentos comprados y consumidos fuera del hogar (por ejemplo, bocaditos, almuerzos de bolsa). Sin embargo, debe excluir las comidas en restaurantes de, y los gastos sobre el alcohol y el tabaco.)

Cuánto gana usted aproximadamente en una semana?

DISPOSICION A PAGAR

Cuánto estarías dispuesto a pagar por semana para completamente eliminar los mosquitos del dengue de tu hogar?

El propósito de este estudio de 16 semanas de investigación cuando nosotros colectamos mosquitos es para explorar nuevos métodos de matar mosquitos para controlar el dengue, específicamente la trampa mata mosquitos que colgamos dentro de su hogar por cuatro semanas. (Muestra la ATSB trampa). Como recordatorio, la trampa de mosquitos está impregnada con agua azucarada y una cantidad mínima de ácido bórico.

Cuánto pagarías por este dispositivo, suponiendo que funciona por un mes y necesitas un dispositivo por cada habitación en su hogar?

PRODUCTOS ANTI-MOSQUITO

Aquí están seis tarjetas con fotos de los productos anti-mosquitos que se pueden encontrar en sus tiendas locales. Estos productos potencialmente pueden reducir el riesgo de dengue en su hogar. (Muestra cada una de las tarjetas y lee una descripción corta).

Cuál de estos productos usted compra ? (Anote la letra correspondiente)	Cuánto gastas en estos productos por semana o por mes?	Con qué frecuencia se utilizan estos productos durante la temporada de dengue (diaria, semanal, mensual)?	Qué factores influyen en su decisión de comprar un producto? (No leer las opciones al principio, permitir la lluvia de ideas libre, entonces sugerir las opciones que no se han mencionado)

(Opciones para la pregunta: (A) Fue recomendado por un familiar/amigo, (B) bajo costo, (C) efectos mínimos en la salud, por ejemplo, problemas respiratorios debido a los productos químicos, (D) de fácil acceso en mi barrio, (E) he utilizado el producto en el pasado y sé que es efectiva, (F) de fácil aplicación/uso, (G) es un nuevo producto que me enteré en la noticia/radio/tv y quiero probarlo, (H) Ministerio de Salud ha recomendado el producto

© 2017 by the authors; licensee MDPI, Basel, Switzerland. This article is an open access article distributed under the terms and conditions of the Creative Commons Attribution (CC-BY) license (http://creativecommons.org/licenses/by/4.0/).
